# Life behind the wall: sensing mechanical cues in plants

**DOI:** 10.1186/s12915-017-0403-5

**Published:** 2017-07-11

**Authors:** Olivier Hamant, Elizabeth S. Haswell

**Affiliations:** 1 Laboratoire Reproduction et Développement des Plantes, University Lyon, ENS de Lyon, UCB Lyon 1, CNRS, INRA, F-69342 Lyon, France; 2 Department of Biology, Washington University in Saint Louis, Mailbox 1137, Saint Louis, MO 63130 USA

## Abstract

There is increasing evidence that all cells sense mechanical forces in order to perform their functions. In animals, mechanotransduction has been studied during the establishment of cell polarity, fate, and division in single cells, and increasingly is studied in the context of a multicellular tissue. What about plant systems? Our goal in this review is to summarize what is known about the perception of mechanical cues in plants, and to provide a brief comparison with animals.

## Plants are pre-stressed structures

**Fig. 1.**
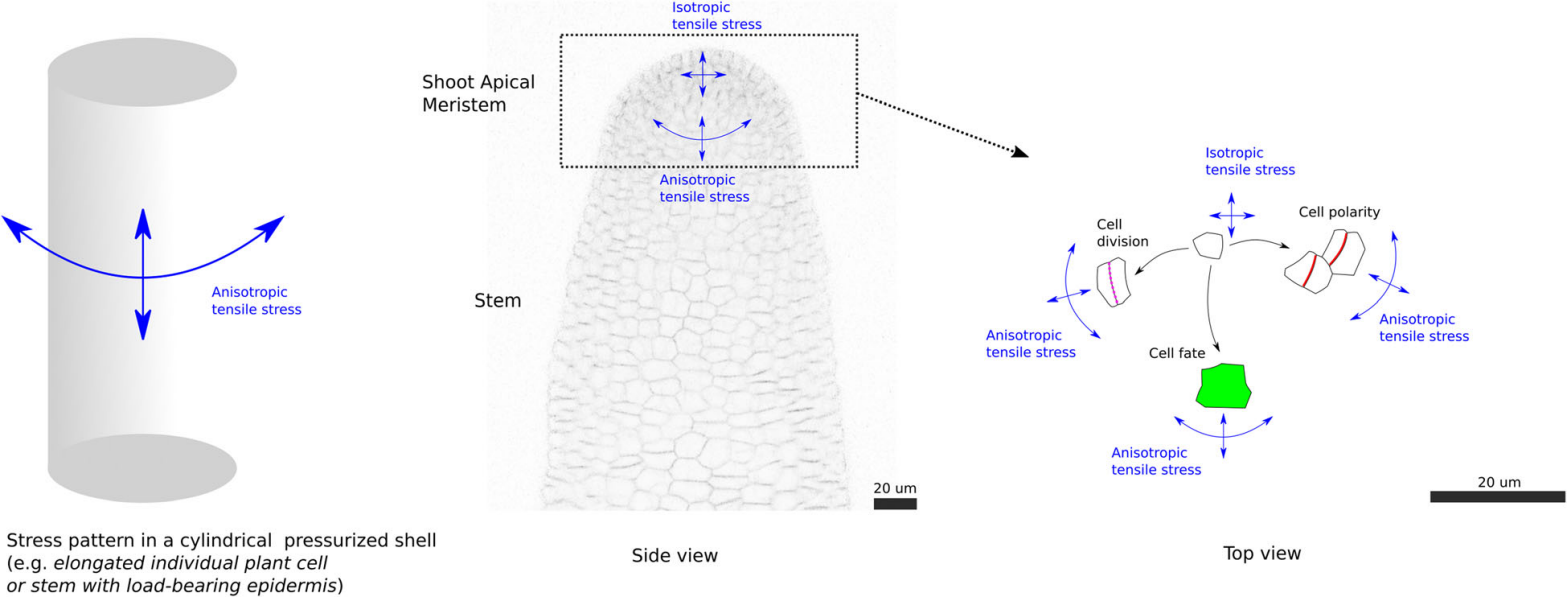
Plants are pre-stressed structures and, in turn, plant cells respond to mechanical cues. **a** Pre-stressed structures are more resilient to mechanical fluctuations and are also energy efficient: a suspension bridge, in which beams are under compression and threads under tension, provides a response to the weak ability of concrete to resist compression, while better allowing swinging and dilatation than an arched bridge. A balloon, with an envelope under tension and a gas under compression, is a pre-stressed structure. When exhibiting a cylindrical shape, such an inflated balloon would display an anisotropic stress pattern, with tension being twice as high in the circumferential direction as in the axial direction. **b** The epidermis of plant aerial organs is under tension, while inner tissues are under compression. Therefore, in the cylindrical stem, tensile stress is predicted to be twice higher transversely than axially. At the apex of the stem, the hemispherical shape of shoot meristem prescribes isotropic tensile stress patterns. Local mechanical conflicts thus arise from cell shape or local differences in growth between adjacent cells. **c** At the shoot apical meristem, as cells are advected away from the meristem center, cells become exposed to varying degrees and direction of mechanical stresses; in turn, such cues can affect cell division plane orientation, gene expression (for example, *STM* expression in *green*) or cell polarity (for example, PIN1 recruitment to the plasma membrane in *red*)

Where does mechanical stress come from in plants? The intrinsic origin of mechanical stress in animals is multifold, from blood hydrodynamics flow [1], muscle deformation [2], or contractile actomyosin cytoskeleton [3]. In plants, if one excludes mechanical perturbations coming from the environment, like the wind, the intrinsic cause of mechanical stress comes down to turgor pressure only [4]. In particular, because plant cells remain glued to each other through their cell walls, they do not migrate or change relative positions. Moreover, in young, growing tissues, cell death does not usually occur. This means that the pattern of stress will derive from pressure stress (shape-derived stress, with cells and tissues modeled as pressure vessels; Fig. 1) and growth-derived stress (turgor pressure being the motor of growth, differential growth and resulting mechanical conflicts will in the end originate from turgor pressure too). Altogether, this means that plant cell biomechanics is much simpler to approach and to model than in animals, as it comes down to solid mechanics, with a balance between turgor pressure and cell wall tension.

Interestingly, the balance between turgor pressure and wall tension at the cell level also scales to the tissue level. There is accumulating evidence that the epidermis plays a load-bearing role in plant development [5], a bit like the cell wall for cell growth. In particular, in the aerial parts of plants, the epidermis often displays thicker cell walls, arguably to provide higher resistance to tension, and in turn revealing where the maximum of tension is. Note that thicker walls may not always be stiffer, as wall stiffness will mainly depend on the composition and texture of the wall. Consistent with an epidermis under tension, peeled epidermises contract. Similarly, superficial cuts in organs usually lead to gap opening [6]. This was notably shown with cuts at the center of sunflower meristems [7] and in *Arabidopsis* cotyledons [8]. The ability of inner tissues to push on the epidermis has also been nicely illustrated with the observation that artificially increasing cell proliferation and cell growth in inner tissues in mutants can generate cracks in the epidermis [9]. Conversely, compressing the shoot apical meristem externally with an indenter results in an elastic response that is more compatible with a pressurized shell than an aggregate of cells [10]. Altogether, plant systems at the cell or tissue level can be considered as pre-stressed systems, with a balance between an envelope (cell wall or epidermis) under tension surrounding content under compression (Fig. 1). This makes the plant system a very competitive model for tissue biomechanics: not only are the mechanical bases of multicellular growth much simpler than in animals, but also the (epidermal) layer that channels morphogenesis is immediately accessible to the microscope.

Here a word of caution needs to be put forward. Internal layers also contribute to morphogenesis, obviously. For instance, vasculature and leaf development are highly coordinated [11] and the contributions of the different epidermal domains in making leaves flat can vary, the marginal meristems being particularly crucial [12, 13]. Furthermore, in roots, the outermost and stiffer layer would be the endodermis, which is located deep inside the tissue and likely plays a role similar to the epidermis in the aerial organs for shaping the root, together with the vasculature [14, 15]. Last, while many molecular regulators of growth are only expressed in the epidermis (for example, the auxin efflux carrier PIN1 is expressed in the epidermis at the shoot apical meristem [16] or the expression of brassinosteroid receptor BRI1 only in the epidermis can rescue the *bri1* mutant [5]), many of these factors can diffuse at the protein or mRNA level, or affect inner layers. There is, for instance, evidence that, while the cytokinin synthesis gene *LOG4* is expressed in the epidermis of the meristem, cytokinin can diffuse inward from that layer, thus impacting other layers [17]. The question one thus needs to address is whether inner layers are following inputs from the epidermis, and whether they can counteract or resist that input. In the case of the cytokinin gradient, it seems that internal layers use this cue to modulate the expression pattern of key regulators of the stem cell niche (*CLV3* and *WUS*) and scale their expression to meristem size [18].

Another word of caution relates to the observation that intrinsic and external mechanical stress cannot be uncoupled so easily in plants. Plants are constantly under mechanical stimulation from their environment, and plants’ final shape is the result of their responses to both internal turgor pressure and external mechanical perturbations [19]. For instance, stem bending can induce a long distance hydraulic pulse that can in turn slow down growth systemically [20].

## Plant cells respond to mechanical signals

One of the first pieces of evidence that plant cells can respond to mechanical stress relates to cell division plane orientation. Several studies have convincingly shown that patterns of cell divisions in plant tissues can be recapitulated with cells dividing along one of the shortest paths [21–23]. When Léo Errera originally stated that plant cells divide along their shortest path (like Oscar Hertwig for animal cells at the same time, [24]), he was using an analogy with soap bubbles [22, 25]. While this rule is often restricted to its geometric terms, the analogy of soap bubbles has mechanical implications, in the form of patterns of surface tension. Following up on those, several studies using mechanical deformations showed that cell division plane orientations could indeed be modified following a change in tensile stress pattern (for example, [26]). By analyzing cell divisions in tissues experiencing mechanical conflicts due to differential growth (the boundary between shoot meristem and organ) and due to tissue shape (circumferential stress being higher in stems because of their cylindrical shape, assuming an epidermis under tension), it was found that cells actually divide along maximal tension, or in other words plant cells build a new wall in the orientation that best resists maximal tensile stress [27] (Fig. 1). Note that mechanical signals may also affect the cell cycle dynamics. This is the subject of many studies in animals, notably in relation to cancer (for example, [28]). While this remains to be investigated in plants, there is some indirect evidence that similar effects could be present. For instance, the highly tensed boundary domain between meristem and organ is also a site of decreased division rate [29], and artificial compression of shoot meristems leads to a transient arrest of cell division [30].

As in animals, the cytoskeleton is also a central feature of the plant cell’s response to mechanical stress. Only a few years after microtubules were discovered (in plants, [31, 32]), Paul Green and colleagues proposed that cortical microtubules align with maximal stress [33]. Conceptually, this is consistent with the alignment of the cell division plane with tensile stress: the cell would reinforce its structure to resist stress, while the tissue would orient its new walls to resist maximal stress. Indeed, by aligning their cortical microtubules with stress, plant cells would guide the trajectory of plasma membrane-localized cellulose synthase complexes, thus reinforcing the wall with cellulose microfibrils in the orientation of maximal stress [33, 34]. Mechanical tests in sunflower hypocotyls [35] and *Arabidopsis* shoot meristems [30] later formally validated that hypothesis. Although actin seems to orient along maximal tensile stress too, this remains poorly documented in plants [36].

Gene expression has also long been known to be under mechanical control in plants. In particular, the developmental response to touch, or thigmomorphogenesis, involves a complete reprogramming of growth, and thus of gene expression. The most famous genes have even been called the *TOUCH* (*TCH*) genes, being induced within minutes after touch [37, 38]. Some of these genes may be involved in signaling. For instance, *TCH3* encodes a calmodulin-related protein, which interacts with the PINOID S/T kinase [39]. Others may also control wall properties, like *TCH4*, which encodes a xyloglucan endotransglucosylase/hydrolase [40]. Yet, most *tch* mutants display wild-type phenotypes, leaving their exact role in the plant response to touch an open question. Two transcription factors associated with mechanical stress have attracted more attention in recent years. In poplar, the expression level of the zinc finger protein *PtaZFP2* has been shown to scale to the extent of stem bending, and thus its expression may in principle be involved in the perception of tissue deformation [41]. At the shoot apical meristem, the promoter activity of the homeobox gene *SHOOT MERISTEMLESS* is enhanced in the boundary domain of the meristem, which is a region of high mechanical stress [42] (Fig. 1). Note that, unlike Twist in *Drosophila* [43–45], *STM* is not expressed ectopically following mechanical perturbations, suggesting that mechanical forces are not the trigger for expression, but rather an additional factor adding robustness to previously established expression patterns.

Last, as in animals, cell polarity is also in part under mechanical control in plants. The recruitment of the auxin efflux carrier PIN1 to the plasma membrane depends on membrane tension in the shoot apical meristem [46], and PIN1 localization to one side of the cell may involve, at least to a certain degree, local differences in tension emerging from differential growth between adjacent cells [47] (Fig. 1). Note, however, that the exact contribution of membrane tension to PIN1 polarity remains to be assessed quantitatively. In particular, the remarkable regularity of organ positions along the stem, despite the presence of cell ablations or microtubule depolymerization at the shoot apical meristem where new organs are initiated, would suggest that PIN1 can become correctly polarized even when the stress pattern is strongly affected. Recently, mechanical forces have also been shown to affect the global pattern of polarity of the BRXL2 protein in leaves [48], showing that planar cell polarity in leaves also include a mechanical contribution, as in the *Drosophila* wing disc for instance [49].

The finding that mechanical forces play an instructive role in cell and developmental biology across kingdoms provides a strong incentive to identify and compare the corresponding mechanotransduction pathways. In plants, the molecular mechanism by which mechanical force informs developing morphology is not yet known. However, a recent accumulation of discoveries regarding mechanoreceptors that perceive internal stimuli such as osmotic swelling or external stimuli such as touch demonstrates that plant mechanoperception can occur at multiple scales. We suspect that at least some of these same mechanisms will be used for developmental purposes as well.

## Plant mechanoperception: the molecular scale

How molecular mechanoreceptors change conformation in response to force is an intriguing but extremely poorly understood biophysical and cell biological problem. Our lack of knowledge is particularly obvious when compared to what we know about the ligand- or light-induced conformational changes that activate photoreceptors or hormone receptors in plants. While the cell wall or the cytoskeleton can be deformed, or deform proteins that are linked to them in response to mechanical force, the complexity of composition and topology of either network makes it difficult to model successfully. Prediction is much simpler at the plasma membrane, where force can act in two constrained dimensions on the membrane in which a protein is embedded. Not coincidentally, the membrane is where our knowledge of mechanosensing in plants is (at the moment) the most advanced.

### Mechanosensitive ion channels

**Fig. 2.**
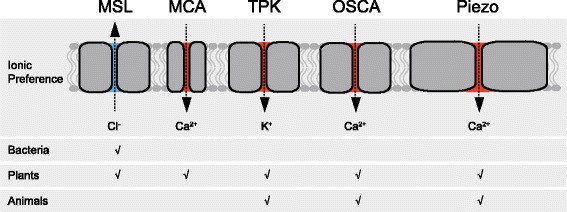
Families of likely plant mechanosensitive ion channels. From *left* to *right*: MscS-like (*MSL*), Mid1-Complementing Activity (*MCA*), Two Pore Potassium (*TPK*), Reduced hyperosmolality-induced [Ca^2+^] increase (*OSCA*), and Piezo channel families, with their proposed primary ion permeability. The presence of homologs in bacterial, plant, and/or animal genomes is indicated with a *checkmark*. The predominant ion flux is shown for each channel, but for simplicity no directionality nor specificity is shown

**Fig. 3.**
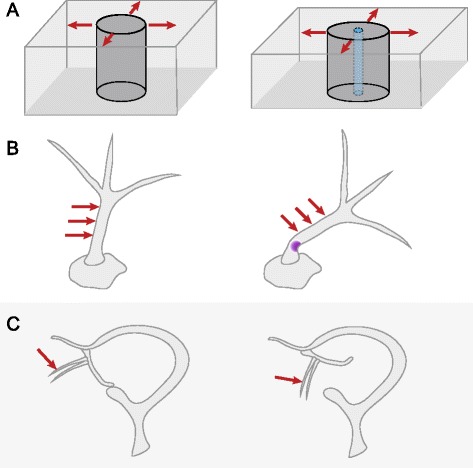
Mechanoreceptors can operate at several distinct scales. **a** Mechanosensitive ion channels provide a clever mechanism to transduce physical force (in the form of lateral membrane tension, *red arrows*) into a change in cellular state (in the form of ion flux). **b** Trichomes, uniquely shaped cells of the plant leaf and stem epidermis, serve as cellular mechanoreceptors by focusing force applied anywhere along the length (*red arrows*) into a buckling movement only at the base (shown in *purple*). **c** Utricularia suction traps may be triggered through a purely mechanical mechanism that relies on a biomechanically bistable structure. Displacement of hairs on the trapdoor leads to rapid opening and closing (not shown) of the trapdoor and any nearby prey is aspirated into the trap

The most conceptually straightforward membrane-bound mechanosensory molecules are mechanosensitive (MS) ion channels (Fig. 2). MS ion channels provide a gated conduit for the passage of ions across a membrane in response to mechanical stimuli. Overviews of MS channel structure, function, and regulation across species can be found in several recent reviews [50–52]. Intrinsically mechanosensitive ion channels are directly opened by increased lateral membrane tension (Fig. 3a); other MS channels may be opened via connections to extracellular or intracellular structures. Once opened, a channel allows ions and other osmolytes to pass across the membrane down their electrochemical gradient. While to some degree all ion channels (and really, all membrane proteins) are mechanosensitive, MS ion channels are distinguished by the fact that their *primary* gating stimulus is force.

Many diverse and evolutionarily unrelated families of MS ion channels have been found in all branches of the tree of life. In plants, MS channels have been proposed to underlie a range of physiological events, including the perception of gravity, vibration, touch, hyper-osmotic and hypo-osmotic stress, pathogenic invasion, interaction with commensal microbes, and pollen tube growth [51, 53–55]. Indeed, Ca^2+^ fluxes and changes in pH have long been correlated with mechanical stimuli [56–58], and recent gene expression analyses reveal the upregulation of predicted MS ion channel genes in response to mechanical signals [59, 60].

Since their initial discovery in plant membranes almost 30 years ago [61–63], over 20 different MS ion channel activities in plant membranes have been identified and characterized to varying degrees (summarized in [50]). It is now clear that MS ion channel activities are widespread in plants from green algae to rice, that MS channels can be expressed in specific cells like leaf mesophyll, guard cells, root cells, and pollen tubes. They also localize to diverse cellular membranes, from the plasma membrane to the vacuolar membrane to the inner membrane of endosymbiotic organelles. Over the past decade, remarkable progress has been made in assigning molecular identity and physiological function to this list of mechanosensitive ion channel activities. At present, five families of MS ion channels or good candidates have been identified in plants (Fig. 2); however, we note that this is unlikely to be an exhaustive list.

The MscS-like, or MSL, channels are homologs of the bacterial MS channel MscS: slightly anion-preferring, intrinsically mechanosensitive channels localized to the mitochondria, plastids, and plasma membrane of a variety of plant tissues. In general, MSL proteins appear to serve as tension-regulated osmotic safety valves [64, 65], although more complex roles have also been suggested [66–68]. The Mid1-complementing activity (MCA) proteins were identified by virtue of their ability to rescue a yeast *Δmid1* phenotype. *MCA* gene overexpression is associated with the increased stretch- and hypo-osmotic shock activated Ca^2+^ influx [69–71] and in *Arabidopsis MCA1* and *MCA2* are redundantly required for root penetration of hard agar [69]. Plant genomes also encode several vacuolar two-pore potassium (TPK) channels that can be mechanically gated during patch clamping [72]. Other likely candidates include the reduced hyperosmolality-induced [Ca^2+^] increase (OSCA)/Calcium-permeable Stress-gated cation Channel (CSC) family, which mediates Ca^2+^ influx in response to increased osmolarity of the extracellular media. Mutants lacking functional *OSCA1* exhibit osmotic-stress related defects [73–75]. Finally, the MS channel Piezo has emerged as a central regulator of many physiological and developmental functions in animals (for example, [76–80]), but nothing has yet been reported for any plant Piezo homologs.

Solid proof that a protein encodes an intrinsically MS ion channel can be elusive. Increased ion flux in response to cell swelling or shrinking is often described as evidence of a mechanosensitive channel at work. However, the timescale of cell volume changes is relatively slow, changes in ionic and osmotic conditions introduce complexity to the interpretation of current, and the channel could still be associated with the cytoskeleton or cell wall. As a result, ion flux in response to swelling/shrinking is not strong evidence for the involvement of an intrinsically mechanosensitive ion channel [52]. Further, linking channel conductance of a candidate mechanosensor to physiological effects requires a range of approaches (not to mention serendipity) and so far has been accomplished for only a few channels in bacteria (MscS, MscL), animals (NOMPC), and plants (MSL8) [81–85].

*Arabidopsis* MSL8 is expressed exclusively in pollen, which harbors the male gametes; and while it is not required for pollen development, it is required for the full survival of pollen grain rehydration and pollen tube growth [65]. Several lines of evidence suggest that the right level of MSL8 activity is crucial: too little and the pollen will not survive swelling during dehydration; too much and the pollen cannot germinate. Presumably MSL8 serves as a release valve for ions, supporting osmotic balance during the extreme challenges of rehydration and germination. Single point mutations in the presumptive pore-lining domain of MSL8 alter both its electrophysiological characteristics and its ability to protect pollen during rehydration and prevent germination when overexpressed, supporting the proposal that the MSL8 channel serves directly as a mechanotransducer.

### Secondary molecular mechanoreceptors

Direct perception through conformational change is only one way for mechanical force to be perceived; it is also possible to perceive the downstream products of mechanical events. For example, several classes of receptor-like kinases (RLKs) may participate indirectly in mechanoperception by detecting cell wall damage. These include, among others, the wall-associated kinases (WAKS) and the *Catharanthus roseus* RLK family (crRLKs) [86, 87]. WAKs possess an extracellular domain capable of binding the pectin backbone and pectin fragments in the cell wall. Genetic evidence indicates they are required for cell expansion during normal development and contribute to pathogen response signaling [88]. Mutations in the crRLK FERONIA result in mechanosensing defects in roots, including an increase in skewing, a defect in penetrating hard agar, and an alteration in the rapid touch-induced cytoplasmic Ca^2+^ signature [89]. FER and other crRLKs such as THESEUS, ANXUR1, and ANXUR2 may sense cell wall integrity via extracellular maltose-binding domains [81, 90]. However, a recent report argues that FER serves as a scaffold to organize other receptors [91]. The latter proposal may explain the observations that the FER kinase activity is not required for mechanosensory or reproductive functions [89, 92], as well as the wide range of pleiotropic phenotypes in the *feronia* mutant [90]. More generally, wall sensing through RLK often goes beyond mechanosensing, and also involves metabolism, biotic and abiotic stresses (for example, [93]). We also note that RLKs could act in synergy with mechanosensitive channels, adding additional complexity to mechanotransduction pathways.

The mechanism by which mechanical signals, both internally and externally derived, are initially perceived at the molecular level is an exciting area of current research at the intersection between physics, cell biology, and engineering [94]. Accordingly, and as can be seen in the research descriptions above, research in this area requires diverse approaches. Molecular genetics in *Arabidopsis thaliana* and *Populus trichocarpa*, live cell imaging, whole cell and excised patch clamp electrophysiology, and mathematical modeling have been used to study mechanobiology. Novel engineering approaches are already helping to overcome the difficulty of controlling mechanical forces, which cannot be manipulated as simply as molecules, and to address the problem of surveying or stimulating the membrane through the thick pectocellulosic plant cell wall. For example, genetically encoded biosensors developed for ions, pH, and small molecules have revolutionized the study of plant cell biology and signal transduction [95–97], and one way to maintain endogenous conditions during mechanobiology experimentation may be the future development of fluorescent biosensors that quantitatively report on physical parameters such as turgor or membrane tension [51, 98].

## Plant mechanoperception at the cellular and organ scales

### Cellular mechanoreceptors

Cells can also serve as mechanoreceptors. As described in Section 2, it is likely that most cells are capable of sensing mechanical signals to coordinate development and maintain shape. “Cellular mechanoreceptors” is thus used here to indicate cells whose *primary* function is to sense a mechanical force. One of the most well-known examples is the trigger hairs of the Venus flytrap. Specialized for touch perception and located on the inner surface of the trap lobes, they sense the presence of an insect or spider, and if the stimulus is repeated, will trigger the closing of the trap [99, 100].

The common trichomes of the *Arabidopsis* leaf surface (shown in Fig. 3b) have recently been shown to serve as unicellular force-focusing mechanoreceptors. Finite element modeling supports a model wherein force applied anywhere along the length of the stiff trichome preferentially results in folding at the base of the trichome (called the “pliant zone”), creating a mechanically sensitive switch. This effect may require a gradient of cell wall thickness, thinnest towards the base [101, 102]. Deformation or brushing of the distal part of a trichome leads to an increase in apoplastic pH and cytosolic Ca^2+^ oscillations in the cells at the base of the trichome, where it joins the epidermis [101]. It is proposed that trichomes may be capable of triggering toxin production or release in response to mechanical signals produced by herbivorous insects; they may also serve as touch receptors during thigmomorphogenesis—the altered developmental program many plants initiate in response to repeated mechanical disturbance [103]. Although trichomes are structurally very different from cilia, their cell geometry serves to amplify the response to external mechanical cues, echoing the function of cilia in response to shear stress or hydrodynamic flow in animal systems (for example, [1]).

### Mechanoreceptor organs

The formation of bistable structures capable of rapid and reversible transition from one state to another (snap-buckling) may allow multicellular structures to serve as mechanoreceptors. While each state is stable, a small input of force sufficient to tip the balance towards the other state will result in a large structural change, often organ movement [104–106]. The trapping mechanism of the aquatic carnivorous bladderwort *Utricularia* spp. may be such a system [107]. The opening of tiny (~1 mm) suction traps of *Utricularia inflata* may be the fastest movement in the plant kingdom, on the order of a millisecond [108].

Under calm conditions, the *Utricularia* suction trap is maintained under negative water pressure, most likely through the pumping action of specialized glands [107, 109]. When prey come into contact with hairs at the mouth of the trap, the door instantly opens, sucking prey along with water into the chamber, and then just as rapidly closes. Modeling experiments as well as observed spontaneous firing events suggest that, due to buckling instability in the trapdoor, only a small disruption of its seal is required to trigger buckling and then full opening of the trapdoor [110–112]. These data support the proposal made by Lloyd in 1929 [113] that the trigger hairs serve only as levers that are pushed by the prey. It remains possible, however, that the hairs instead serve as physiological sensors capable of inducing the propagation of an electrical signal and the closing of a bistable trap (as do the trigger hairs of the Venus flytrap [97]) but few data exist to support this proposal [107, 111].

## Plants and animal mechanoperception—similar but different

To conclude, while both plants and animals respond to mechanical stimuli for their development and physiology, their mechanosensing pathways are only partially homologous. The differences may be due to substantial quantitative difference in stress levels: osmotic pressure in animal cells is in the kPa range while plant cells experience turgor pressure in the MPa range. Divergence in mechanosensing pathways may also relate to qualitative differences in the growth and deformation strategies of plants and animals: because of the presence of a stiff cell wall in plants, deformation in plants is triggered either by growth or by (potentially rapid) changes in turgor pressure. In contrast, deformation and the corresponding changes in stress patterns in animals usually comes down to contractility-related mechanisms. This idea is supported by the apparent absence of focal adhesion proteins such as vinculin and actinin, and Yap/Taz proteins in plants. On the other hand, the ownership of a plasma membrane is a fundamental characteristic of a cell, and correspondingly MS channels may provide a universal approach to force perception [114].

Yet, the questions of how endogenous mechanical forces are sensed and the threshold beyond which a response to mechanical signals is triggered largely remain unanswered, notably because of the lack of quantitative approaches to measure stress levels at subcellular scales. The current development of FRET-based tension sensors is an important step in that direction. In the future, it might be possible to determine the threshold beyond which channels and mechanosensors are activated, and the threshold beyond which the cell response to such signals is amplified. This may very well help us understand how cells discriminate between mechanical noise and mechanical signals, an important question both for signals that originate inside and for those that originate outside the cell wall.

## References

[1] Heckel E, Boselli F, Roth S, Krudewig A, Belting H-G, Charvin G (2015). Oscillatory flow modulates mechanosensitive klf2a expression through trpv4 and trpp2 during heart valve development. Curr Biol.

[2] Kahn J, Shwartz Y, Blitz E, Krief S, Sharir A, Breitel DA (2009). Muscle contraction is necessary to maintain joint progenitor cell fate. Dev Cell.

[3] Lecuit T, Lenne P-F (2007). Cell surface mechanics and the control of cell shape, tissue patterns and morphogenesis. Nat Rev Mol Cell Biol.

[4] Peters WS, Tomos AD (1996). The history of tissue tension. Ann Bot.

[5] Savaldi-Goldstein S, Peto C, Chory J (2007). The epidermis both drives and restricts plant shoot growth. Nature.

[6] Kutschera U, Niklas KJ (2007). The epidermal-growth-control theory of stem elongation: an old and a new perspective. J Plant Physiol.

[7] Dumais J, Steele CR (2000). New evidence for the role of mechanical forces in the shoot apical meristem. J Plant Growth Regul.

[8] Sampathkumar A, Krupinski P, Wightman R, Milani P, Berquand A, Boudaoud A (2014). Subcellular and supracellular mechanical stress prescribes cytoskeleton behavior in Arabidopsis cotyledon pavement cells. elife.

[9] Maeda S, Gunji S, Hanai K, Hirano T, Kazama Y, Ohbayashi I (2014). The conflict between cell proliferation and expansion primarily affects stem organogenesis in Arabidopsis. Plant Cell Physiol.

[10] Beauzamy L, Louveaux M, Hamant O, Boudaoud A (2015). Mechanically, the shoot apical meristem of arabidopsis behaves like a shell inflated by a pressure of about 1 MPa. Front Plant Sci.

[11] Scarpella E, Barkoulas M, Tsiantis M (2010). Control of leaf and vein development by auxin. Cold Spring Harb Perspect Biol.

[12] Alvarez JP, Furumizu C, Efroni I, Eshed Y, Bowman JL (2016). Active suppression of a leaf meristem orchestrates determinate leaf growth. elife.

[13] Nath U, Crawford BCW, Carpenter R, Coen E (2003). Genetic control of surface curvature. Science.

[14] Péret B, Li G, Zhao J, Band LR, Voß U, Postaire O (2012). Auxin regulates aquaporin function to facilitate lateral root emergence. Nat Cell Biol.

[15] Vermeer JEM, von Wangenheim D, Barberon M, Lee Y, Stelzer EHK, Maizel A (2014). A spatial accommodation by neighboring cells is required for organ initiation in Arabidopsis. Science.

[16] Reinhardt D, Pesce E-R, Stieger P, Mandel T, Baltensperger K, Bennett M (2003). Regulation of phyllotaxis by polar auxin transport. Nature.

[17] Chickarmane VS, Gordon SP, Tarr PT, Heisler MG, Meyerowitz EM (2012). Cytokinin signaling as a positional cue for patterning the apical-basal axis of the growing Arabidopsis shoot meristem. Proc Natl Acad Sci U S A.

[18] Gruel J, Landrein B, Tarr P, Schuster C, Refahi Y, Sampathkumar A (2016). An epidermis-driven mechanism positions and scales stem cell niches in plants. Sci Adv.

[19] Moulia B, Coutand C, Julien J-L (2015). Mechanosensitive control of plant growth: bearing the load, sensing, transducing, and responding. Front Plant Sci.

[20] Lopez R, Badel E, Peraudeau S, Leblanc-Fournier N, Beaujard F, Julien J-L (2014). Tree shoot bending generates hydraulic pressure pulses: a new long-distance signal?. J Exp Bot.

[21] Dupuy L, Mackenzie J, Haseloff J (2010). Coordination of plant cell division and expansion in a simple morphogenetic system. Proc Natl Acad Sci U S A.

[22] Besson S, Dumais J (2011). Universal rule for the symmetric division of plant cells. Proc Natl Acad Sci U S A.

[23] Yoshida S, Barbier de Reuille P, Lane B, Bassel GW, Prusinkiewicz P, Smith RS (2014). Genetic control of plant development by overriding a geometric division rule. Dev Cell.

[24] Hertwig O. Das Problem der Befruchtung und der Isotropie des Eies. Eine Theorie der Vererbung. Jenaische Z. Naturwissenschaft. 1884;274.

[25] Errera L. Sur une condition fondamentale d’e’ quilibre des cellules vivantes. C R Hebd Seances Acad Sci. 1886;822–4.

[26] Lintilhac PM, Vesecky TB (1984). Stress-induced alignment of division plane in plant tissues grown in vitro. Nature.

[27] Louveaux M, Julien J-D, Mirabet V, Boudaoud A, Hamant O (2016). Cell division plane orientation based on tensile stress in Arabidopsis thaliana. Proc Natl Acad Sci U S A.

[28] Fernández-Sánchez ME, Barbier S, Whitehead J, Béalle G, Michel A, Latorre-Ossa H (2015). Mechanical induction of the tumorigenic β-catenin pathway by tumour growth pressure. Nature.

[29] Breuil-Broyer S, Morel P, de Almeida-Engler J, Coustham V, Negrutiu I, Trehin C (2004). High-resolution boundary analysis during Arabidopsis thaliana flower development. Plant J Cell Mol Biol.

[30] Hamant O, Heisler MG, Jonsson H, Krupinski P, Uyttewaal M, Bokov P (2008). Developmental patterning by mechanical signals in Arabidopsis. Science.

[31] Ledbetter MC, Porter KR (1963). A “microtubule” in plant cell fine structure. J Cell Biol.

[32] Green PB (1962). Mechanism for plant cellular morphogenesis. Science.

[33] Green P, King A. A mechanism for the origin of specifically oriented textures in development with special reference to Nitella wall texture. Aust J Biol Sci. 1966;421–37

[34] Williamson R. Alignment of cortical microtubules by anisotropic wall stresses. Aust J Plant Physiol. 1990;601–13.

[35] Hejnowicz Z, Rusin A, Rusin T (2000). Tensile tissue stress affects the orientation of cortical microtubules in the epidermis of sunflower hypocotyl. J Plant Growth Regul.

[36] Goodbody KC, Lloyd CW (1990). Actin filaments line up acrossTradescantia epidermal cells, anticipating wound-induced divison planes. Protoplasma.

[37] Braam J (2005). In touch: plant responses to mechanical stimuli. New Phytol.

[38] Lee D, Polisensky DH, Braam J (2005). Genome-wide identification of touch- and darkness-regulated Arabidopsis genes: a focus on calmodulin-like and XTH genes. New Phytol.

[39] Benjamins R, Ampudia CSG, Hooykaas PJJ, Offringa R (2003). PINOID-mediated signaling involves calcium-binding proteins. Plant Physiol.

[40] Xu W, Purugganan MM, Polisensky DH, Antosiewicz DM, Fry SC, Braam J (1995). Arabidopsis TCH4, regulated by hormones and the environment, encodes a xyloglucan endotransglycosylase. Plant Cell.

[41] Coutand C, Martin L, Leblanc-Fournier N, Decourteix M, Julien J-L, Moulia B (2009). Strain mechanosensing quantitatively controls diameter growth and PtaZFP2 gene expression in poplar. Plant Physiol.

[42] Landrein B, Kiss A, Sassi M, Chauvet A, Das P, Cortizo M (2015). Mechanical stress contributes to the expression of the STM homeobox gene in Arabidopsis shoot meristems. elife.

[43] Farge E (2003). Mechanical induction of Twist in the Drosophila foregut/stomodeal primordium. Curr Biol.

[44] Desprat N, Supatto W, Pouille P-A, Beaurepaire E, Farge E (2008). Tissue deformation modulates twist expression to determine anterior midgut differentiation in Drosophila embryos. Dev Cell.

[45] Brunet T, Bouclet A, Ahmadi P, Mitrossilis D, Driquez B, Brunet A-C (2013). Evolutionary conservation of early mesoderm specification by mechanotransduction in Bilateria. Nat Commun.

[46] Nakayama N, Smith RS, Mandel T, Robinson S, Kimura S, Boudaoud A (2012). Mechanical regulation of auxin-mediated growth. Curr Biol.

[47] Heisler MG, Hamant O, Krupinski P, Uyttewaal M, Ohno C, Jonsson H (2010). Alignment between PIN1 polarity and microtubule orientation in the shoot apical meristem reveals a tight coupling between morphogenesis and auxin transport. PLoS Biol.

[48] Bringmann M, Bergmann DC (2017). Tissue-wide mechanical forces influence the polarity of stomatal stem cells in Arabidopsis. Curr Biol.

[49] Aigouy B, Farhadifar R, Staple DB, Sagner A, Röper J-C, Jülicher F (2010). Cell flow reorients the axis of planar polarity in the wing epithelium of Drosophila. Cell.

[50] Hamilton ES, Schlegel AM, Haswell ES (2015). United in diversity: mechanosensitive ion channels in plants. Annu Rev Plant Biol.

[51] Peyronnet R, Tran D, Girault T, Frachisse J-M (2014). Mechanosensitive channels: feeling tension in a world under pressure. Front Plant Sci.

[52] Ranade SS, Syeda R, Patapoutian A (2015). Mechanically activated ion channels. Neuron.

[53] Toyota M, Gilroy S (2013). Gravitropism and mechanical signaling in plants. Am J Bot.

[54] Jayaraman D, Gilroy S, Ané J-M (2014). Staying in touch: mechanical signals in plant-microbe interactions. Curr Opin Plant Biol.

[55] Haswell ES, Verslues PE (2015). The ongoing search for the molecular basis of plant osmosensing. J Gen Physiol.

[56] Trewavas A, Knight M (1994). Mechanical signalling, calcium and plant form. Plant Mol Biol.

[57] Fasano JM, Massa GD, Gilroy S (2002). Ionic signaling in plant responses to gravity and touch. J Plant Growth Regul.

[58] Monshausen GB, Haswell ES (2013). A force of nature: molecular mechanisms of mechanoperception in plants. J Exp Bot.

[59] Ghosh R, Gururani MA, Ponpandian LN, Mishra RC, Park S-C, Jeong M-J (2017). Expression analysis of sound vibration-regulated genes by touch treatment in Arabidopsis. Front Plant Sci.

[60] uuml lse de Silvia GHS, da Adriana PS, Tania MI, da Eduardo GS, uacute Talita C (2016). Genome-wide analysis of mechanosensitive channel of small conductance (MscS)-like gene family in common bean. Afr J Biotechnol.

[61] Falke LC, Edwards KL, Pickard BG, Misler S (1988). A stretch-activated anion channel in tobacco protoplasts. FEBS Lett.

[62] Cosgrove DJ, Hedrich R (1991). Stretch-activated chloride, potassium, and calcium channels coexisting in plasma membranes of guard cells of Vicia faba L. Planta.

[63] Schroeder JI, Hedrich R (1989). Involvement of ion channels and active transport in osmoregulation and signaling of higher plant cells. Trends Biochem Sci.

[64] Veley KM, Marshburn S, Clure CE, Haswell ES (2012). Mechanosensitive channels protect plastids from hypoosmotic stress during normal plant growth. Curr Biol.

[65] Hamilton ES, Jensen GS, Maksaev G, Katims A, Sherp AM, Haswell ES (2015). Mechanosensitive channel MSL8 regulates osmotic forces during pollen hydration and germination. Science.

[66] Zhang Z, Tateda C, Jiang S-C, Shrestha J, Jelenska J, Speed DJ (2017). A suite of receptor-like kinases and a putative mechano-sensitive channel are involved in autoimmunity and plasma membrane-based defenses in Arabidopsis. Mol Plant-Microbe Interact MPMI.

[67] Veley KM, Maksaev G, Frick EM, January E, Kloepper SC, Haswell ES (2014). Arabidopsis MSL10 has a regulated cell death signaling activity that is separable from its mechanosensitive ion channel activity. Plant Cell.

[68] Lee CP, Maksaev G, Jensen GS, Murcha MW, Wilson ME, Fricker M (2016). MSL1 is a mechanosensitive ion channel that dissipates mitochondrial membrane potential and maintains redox homeostasis in mitochondria during abiotic stress. Plant J Cell Mol Biol.

[69] Nakagawa Y, Katagiri T, Shinozaki K, Qi Z, Tatsumi H, Furuichi T (2007). Arabidopsis plasma membrane protein crucial for Ca2+ influx and touch sensing in roots. Proc Natl Acad Sci U S A.

[70] Kurusu T, Yamanaka T, Nakano M, Takiguchi A, Ogasawara Y, Hayashi T (2012). Involvement of the putative Ca^2+^-permeable mechanosensitive channels, NtMCA1 and NtMCA2, in Ca^2+^ uptake, Ca^2+^-dependent cell proliferation and mechanical stress-induced gene expression in tobacco (Nicotiana tabacum) BY-2 cells. J Plant Res.

[71] Kurusu T, Nishikawa D, Yamazaki Y, Gotoh M, Nakano M, Hamada H (2012). Plasma membrane protein OsMCA1 is involved in regulation of hypo-osmotic shock-induced Ca2+ influx and modulates generation of reactive oxygen species in cultured rice cells. BMC Plant Biol.

[72] Maathuis FJM (2011). Vacuolar two-pore K+ channels act as vacuolar osmosensors. New Phytol.

[73] Yuan F, Yang H, Xue Y, Kong D, Ye R, Li C (2014). OSCA1 mediates osmotic-stress-evoked Ca2+ increases vital for osmosensing in Arabidopsis. Nature.

[74] Li Y, Yuan F, Wen Z, Li Y, Wang F, Zhu T (2015). Genome-wide survey and expression analysis of the OSCA gene family in rice. BMC Plant Biol.

[75] Hou C, Tian W, Kleist T, He K, Garcia V, Bai F (2014). DUF221 proteins are a family of osmosensitive calcium-permeable cation channels conserved across eukaryotes. Cell Res.

[76] Ranade SS, Qiu Z, Woo S-H, Hur SS, Murthy SE, Cahalan SM (2014). Piezo1, a mechanically activated ion channel, is required for vascular development in mice. Proc Natl Acad Sci U S A.

[77] Li J, Hou B, Tumova S, Muraki K, Bruns A, Ludlow MJ (2014). Piezo1 integration of vascular architecture with physiological force. Nature.

[78] Koser DE, Thompson AJ, Foster SK, Dwivedy A, Pillai EK, Sheridan GK (2016). Mechanosensing is critical for axon growth in the developing brain. Nat Neurosci.

[79] Nonomura K, Woo S-H, Chang RB, Gillich A, Qiu Z, Francisco AG (2016). Piezo2 senses airway stretch and mediates lung inflation-induced apnoea. Nature.

[80] Gudipaty SA, Lindblom J, Loftus PD, Redd MJ, Edes K, Davey CF (2017). Mechanical stretch triggers rapid epithelial cell division through Piezo1. Nature.

[81] Edwards MD, Li Y, Kim S, Miller S, Bartlett W, Black S (2005). Pivotal role of the glycine-rich TM3 helix in gating the MscS mechanosensitive channel. Nat Struct Mol Biol.

[82] Yan Z, Zhang W, He Y, Gorczyca D, Xiang Y, Cheng LE (2013). Drosophila NOMPC is a mechanotransduction channel subunit for gentle-touch sensation. Nature.

[83] Kang L, Gao J, Schafer WR, Xie Z, Xu XZS (2010). C. elegans TRP family protein TRP-4 is a pore-forming subunit of a native mechanotransduction channel. Neuron.

[84] Hamilton ES, Haswell ES (2017). The tension-sensitive ion transport activity of MSL8 is critical for its function in pollen hydration and germination. Plant Cell Physiol.

[85] Blount P, Schroeder MJ, Kung C (1997). Mutations in a bacterial mechanosensitive channel change the cellular response to osmotic stress. J Biol Chem.

[86] Engelsdorf T, Hamann T (2014). An update on receptor-like kinase involvement in the maintenance of plant cell wall integrity. Ann Bot.

[87] Wolf S, Hématy K, Höfte H (2012). Growth control and cell wall signaling in plants. Annu Rev Plant Biol.

[88] Kohorn BD (2015). The state of cell wall pectin monitored by wall associated kinases: A model. Plant Signal Behav.

[89] Shih H-W, Miller ND, Dai C, Spalding EP, Monshausen GB (2014). The receptor-like kinase FERONIA is required for mechanical signal transduction in Arabidopsis seedlings. Curr Biol.

[90] Li C, Wu H-M, Cheung AY (2016). FERONIA and her pals: functions and mechanisms. Plant Physiol.

[91] Stegmann M, Monaghan J, Smakowska-Luzan E, Rovenich H, Lehner A, Holton N (2017). The receptor kinase FER is a RALF-regulated scaffold controlling plant immune signaling. Science.

[92] Kessler SA, Lindner H, Jones DS, Grossniklaus U (2015). Functional analysis of related CrRLK1L receptor-like kinases in pollen tube reception. EMBO Rep.

[93] Van der Does D, Boutrot F, Engelsdorf T, Rhodes J, McKenna JF, Vernhettes S (2017). The Arabidopsis leucine-rich repeat receptor kinase MIK2/LRR-KISS connects cell wall integrity sensing, root growth and response to abiotic and biotic stresses. PLoS Genet.

[94] Moulia B (2013). Plant biomechanics and mechanobiology are convergent paths to flourishing interdisciplinary research. J Exp Bot.

[95] Uslu VV, Grossmann G (2016). The biosensor toolbox for plant developmental biology. Curr Opin Plant Biol.

[96] Okumoto S, Jones A, Frommer WB (2012). Quantitative imaging with fluorescent biosensors. Annu Rev Plant Biol.

[97] Choi W-G, Swanson SJ, Gilroy S (2012). High-resolution imaging of Ca2+, redox status, ROS and pH using GFP biosensors. Plant J Cell Mol Biol.

[98] Freikamp A, Mehlich A, Klingner C, Grashoff C (2017). Investigating piconewton forces in cells by FRET-based molecular force microscopy. J Struct Biol.

[99] Böhm J, Scherzer S, Krol E, Kreuzer I, von Meyer K, Lorey C (2016). The Venus flytrap Dionaea muscipula counts prey-induced action potentials to induce sodium uptake. Curr Biol.

[100] DiPalma JR, McMichael R, DiPalma M (1966). Touch receptor of venous flytrap, Dionaea muscipula. Science.

[101] Zhou LH, Liu SB, Wang PF, Lu TJ, Xu F, Genin GM (2017). The Arabidopsis trichome is an active mechanosensory switch. Plant Cell Environ.

[102] Liu H, Zhou LH, Jiao J, Liu S, Zhang Z, Lu TJ (2016). Gradient mechanical properties facilitate arabidopsis trichome as mechanosensor. ACS Appl Mater Interfaces.

[103] Chehab EW, Eich E, Braam J (2009). Thigmomorphogenesis: a complex plant response to mechano-stimulation. J Exp Bot.

[104] Chen Z, Guo Q, Majidi C, Chen W, Srolovitz DJ, Haataja MP (2012). Nonlinear geometric effects in mechanical bistable morphing structures. Phys Rev Lett.

[105] Li S, Wang KW (2016). Plant-inspired adaptive structures and materials for morphing and actuation: a review. Bioinspir Biomim.

[106] Noblin X, Rojas NO, Westbrook J, Llorens C, Argentina M, Dumais J (2012). The fern sporangium: a unique catapult. Science.

[107] Poppinga S, Weisskopf C, Westermeier AS, Masselter T, Speck T (2015). Fastest predators in the plant kingdom: functional morphology and biomechanics of suction traps found in the largest genus of carnivorous plants. AoB PLANTS.

[108] Vincent O, Weisskopf C, Poppinga S, Masselter T, Speck T, Joyeux M (2011). Ultra-fast underwater suction traps. Proc Biol Sci.

[109] Sydenham PH, Findlay GP. Transport of solutes and water by resetting bladders of Utricularia. Aust J Plant Physiol. 1975;335–51.

[110] Adamec L. Firing and resetting characteristics of carnivorous Utricularia reflexa traps: physiological or only physical regulation of trap triggering? Phyton Horn Austria. 2012;281–90.

[111] Adamec L (2011). The smallest but fastest: ecophysiological characteristics of traps of aquatic carnivorous Utricularia. Plant Signal Behav.

[112] Vincent O, Roditchev I, Marmottant P (2011). Spontaneous firings of carnivorous aquatic Utricularia traps: temporal patterns and mechanical oscillations. PLoS One.

[113] Lloyd FE (1929). The mechanism of the water tight door of the Utricularia trap. Plant Physiol.

[114] Anishkin A, Loukin SH, Teng J, Kung C (2014). Feeling the hidden mechanical forces in lipid bilayer is an original sense. Proc Natl Acad Sci U S A.

